# Supplementary material for: Testis single-cell RNA-seq reveals the dynamics of de novo gene transcription and germline mutational bias in Drosophila
Source: eLife. 2019 Aug 16;8:e47138. doi: 10.7554/eLife.47138 (PMC6697446; doi:10.7554/eLife.47138)
Supplement: Transparent reporting form [file elife-47138-transrepform.docx]

***eLife’s* transparent reporting form**

We encourage authors to provide detailed information *within their submission* to facilitate the interpretation and replication of experiments. Authors can upload supporting documentation to indicate the use of appropriate reporting guidelines for health-related research (see [EQUATOR Network](http://www.equator-network.org/%20)), life science research (see the [BioSharing Information Resource](https://biosharing.org/)), or the [ARRIVE guidelines](http://www.plosbiology.org/article/info:doi/10.1371/journal.pbio.1000412) for reporting work involving animal research. Where applicable, authors should refer to any relevant reporting standards documents in this form.

If you have any questions, please consult our Journal Policies and/or contact us: [editorial@elifesciences.org](mailto:editorial@elifesciences.org).

**Sample-size estimation**

- You should state whether an appropriate sample size was computed when the study was being designed
- You should state the statistical method of sample size computation and any required assumptions
- If no explicit power analysis was used, you should describe how you decided what sample (replicate) size (number) to use

Please outline where this information can be found within the submission (e.g., sections or figure legends), or explain why this information doesn’t apply to your submission:

We sequenced 5000 cells to ensure that we captured at least 50,000 reads per cell. On average, we generated about 100,000 reads per cell, which is exceeds the norm of current publish standard. We also correlated the single-cell RNA-seq data with bulk RNA-seq data from the same strain as well as another single-cell RNA-seq data from testis of a wild types strain.

**Replicates**

- You should report how often each experiment was performed
- You should include a definition of biological versus technical replication
- The data obtained should be provided and sufficient information should be provided to indicate the number of independent biological and/or technical replicates
- If you encountered any outliers, you should describe how these were handled
- Criteria for exclusion/inclusion of data should be clearly stated
- High-throughput sequence data should be uploaded before submission, with a private link for reviewers provided (these are available from both GEO and ArrayExpress)

Please outline where this information can be found within the submission (e.g., sections or figure legends), or explain why this information doesn’t apply to your submission:

For single-cell sequencing, biological replicates are unfeasible due to the enormous cost of library preparation and additional sequencing. For statistical purposes such as the calculation of gene expression within our library, each of the 5000 cells can be considered a single replicate. To estimate the quality and reproducibility of our dataset, we correlated the single-cell RNA-seq data with bulk RNA-seq data from the same strain as well as another single-cell RNA-seq data from testis of a wild type strain.

**Statistical reporting**

- Statistical analysis methods should be described and justified
- Raw data should be presented in figures whenever informative to do so (typically when N per group is less than 10)
- For each experiment, you should identify the statistical tests used, exact values of N, definitions of center, methods of multiple test correction, and dispersion and precision measures (e.g., mean, median, SD, SEM, confidence intervals; and, for the major substantive results, a measure of effect size (e.g., Pearson's r, Cohen's d)
- Report exact p-values wherever possible alongside the summary statistics and 95% confidence intervals. These should be reported for all key questions and not only when the p-value is less than 0.05.

Please outline where this information can be found within the submission (e.g., sections or figure legends), or explain why this information doesn’t apply to your submission:

Supplemental table 3 contains all raw and Hochberg-adjusted p values for gene bias calculations. Figure 5 includes 95 percent confidence intervals for a chi-square proportion test. We reported the statistical methods for the p values and quantitative measures included in the manuscript.

(For large datasets, or papers with a very large number of statistical tests, you may upload a single table file with tests, Ns, etc., with reference to sections in the manuscript.)

**Group allocation**

- Indicate how samples were allocated into experimental groups (in the case of clinical studies, please specify allocation to treatment method); if randomization was used, please also state if restricted randomization was applied
- Indicate if masking was used during group allocation, data collection and/or data analysis

Please outline where this information can be found within the submission (e.g., sections or figure legends), or explain why this information doesn’t apply to your submission:

Our analysis takes place on one group of an isogenic strain of *D. melanogaster*.

**Additional data files (“source data”)**

- We encourage you to upload relevant additional data files, such as numerical data that are represented as a graph in a figure, or as a summary table
- Where provided, these should be in the most useful format, and they can be uploaded as “Source data” files linked to a main figure or table
- Include model definition files including the full list of parameters used
- Include code used for data analysis (e.g., R, MatLab)
- Avoid stating that data files are “available upon request”

Please indicate the figures or tables for which source data files have been provided:

Fastq files of the single-cell testis RNA-seq data have been deposited at NCBI SRA with accession number PRJNA517685 and PRJNA548742.

Lists of genes used for gene bias calculations are found in Supplemental Table 6.

All variants and their cell types are detailed in Supplemental Figure 5.

R code for the construction of a custom cellranger reference was deposited to github, linked in the manuscript.
